# Impact of Medicaid Continuous Glucose Monitor Expansion on Uptake, Glycemic Outcomes, and Engagement Among Adults With Type 2 Diabetes in a Federally Qualified Health Center

**DOI:** 10.1177/21501319261433345

**Published:** 2026-04-20

**Authors:** Walter Solorzano, Ligaya Docena Scarlett, Giuliana Perini Villanueva, Kathyana Santiago Mangual, Cynthia Santana, Bryan Escobar, Angelique Rubio, Nicholas J Jackson, Marielle Tavares, Joseph Borrell, Beatrice Brumley, Tannaz Moin, Estelle Everett

**Affiliations:** 1 University of California, Los Angeles, USA; 2 Venice Family Clinic, Los Angeles, CA, USA; 3 VA Greater Los Angeles Healthcare System, CA, USA

**Keywords:** continuous glucose monitoring, type 2 diabetes, Medicaid policy, health equity, safety-net healthcare, federally qualified health centers

## Abstract

**Background:**

Continuous glucose monitors (CGMs) improve outcomes for adults with type 2 diabetes yet use remains low in underserved populations. California’s 2022 Medicaid coverage expansion aimed to reduce access barriers, but its impact in safety-net settings is not well understood.

**Methods:**

We conducted a retrospective cohort study of adults with type 2 diabetes receiving care at a federally qualified health center from January 2022 to June 2023. Electronic health records assessed CGM uptake and glycemic outcomes. Telephone surveys (November 2023-February 2025) evaluated patient perceptions and engagement. Time-to-event models estimated the association between CGM initiation and achieving a ≥1% hemoglobin A1c (HbA1c) reduction. Open-ended responses were analyzed thematically.

**Results:**

Among 256 adults, 126 (49%) initiated CGM use, with uptake increasing after Medicaid expansion. In the analytic sample (n = 230), CGM new users were associated with a higher but not statistically significant hazard of achieving a ≥1% HbA1c reduction (adjusted HR = 1.31; 95% CI: 0.87-1.97). Among survey respondents (n = 82), commonly cited benefits included improved glucose management and convenience, while engagement varied across behaviors.

**Conclusions:**

Medicaid coverage expansion increased CGM use in a safety-net population. High acceptability alongside variable engagement highlights the need for targeted implementation strategies to translate expanded access into equitable clinical benefit.

## Introduction

Continuous glucose monitors (CGMs) have transformed diabetes management by providing real-time glucose data, hypoglycemia and hyperglycemia alerts, and trend information that enhances both self-management and clinical decision-making.^1,2^ Evidence demonstrates that CGM use is associated with improved glycosylated hemoglobin (HbA1c), reduced diabetes-related distress and fear of hypoglycemia,^3-5^ greater adherence to dietary and physical activity recommendations,^6-9^ and improved glycemic control and treatment satisfaction among adults with insulin-treated type 2 diabetes.^10-13^

Despite these advantages, substantial disparities in CGM use persist, particularly among publicly insured, lower-income, and racially and ethnically minoritized populations.^14-17^ Prior CGM disparities research has largely focused on individuals with type 1 diabetes.^14-17^ Although emerging work has examined CGM use among adults with type 2 diabetes in safety-net settings, which are health systems that provide a substantial proportion of care to Medicaid-insured, uninsured, and other socioeconomically vulnerable populations, including federally qualified health centers and public hospital systems,^18^ little is known about how patients in these settings adopt and engage with CGM technology.

Insurance-related restrictions have historically limited CGM access for adults with type 2 diabetes. Publicly insured patients have 57% lower odds of being prescribed a CGM,^19^ and recent national analyses of federally qualified health centers (FQHCs) show CGM prescribing rates of only ~1%.^18^ These findings suggest that beyond prescribing disparities, real-world CGM use remains limited in safety-net settings serving predominantly publicly insured populations. Restrictive eligibility requirements and coverage policies have further constrained access among Medicaid beneficiaries compared with commercially insured patients.^19^ In January 2022, California implemented a major policy change by expanding Medicaid coverage of CGMs to all insulin-treated individuals and changed to a pharmacy benefit under Medi-Cal Rx, replacing the prior durable medical equipment approval pathway.^20^ However, evidence describing how this policy change influenced CGM uptake, glycemic outcomes, and patient experiences in safety-net clinical settings remains limited.

This study examined CGM uptake and glycemic outcomes among adults with type 2 diabetes following California’s Medicaid policy expansion. In addition, we evaluated patient-reported perceptions of CGM use, including perceived benefits, drawbacks, and day-to-day engagement, to characterize real-world experiences with CGM technology in a resource-limited setting. By integrating chart review and patient-reported data, this study addresses a critical gap in understanding how adults with type 2 diabetes in the safety net adopt, engage with, and perceive CGM technology following Medicaid coverage expansion.

## Methods

This retrospective cohort study evaluated the impact of California’s January 2022 Medicaid CGM coverage expansion on CGM uptake, glycemic outcomes, and patient-reported experiences among adults with type 2 diabetes receiving care at a federally qualified health center (FQHC). Electronic health record (EHR) data were extracted for eligible patients seen between January 1, 2022, and June 30, 2023. Patient surveys were administered between November 2023 and February 2025 and included open-ended questions on CGM perceptions and Likert-scale items assessing CGM engagement.

### Setting

The study was conducted at a multi-site federally qualified health center in an urban safety-net setting serving a predominantly publicly insured population. The health center operates a Diabetes Management Program (DMP) that provides intensive, multidisciplinary care for adults with poorly controlled diabetes. Within the DMP, nurse practitioners serve as the primary clinicians and provide longitudinal diabetes management, including medication titration and CGM prescribing.

Prior to and throughout the study period, the DMP maintained structured support for CGM initiation, including same-day device starts through on-site pharmacies and hands-on onboarding by bilingual health educators. Health educators assisted with app setup, sensor application, troubleshooting, and routine follow-up every 10 to 14 days. Following the January 2022 policy change, expanded insurance eligibility allowed a substantially greater number of patients to access these existing services.

### Sample

Adults were eligible if they had (1) type 2 diabetes, (2) any insulin use during the study period, and (3) at least 2 visits with a DMP nurse practitioner between January 1, 2022, and June 30, 2023. Patients without insulin use at any point during the study window were excluded because they were ineligible for CGM coverage. Insulin status was determined based on use at any time during the study period; some patients were subsequently transitioned off insulin during follow-up as part of routine clinical care. For the survey, we invited all adults with documented CGM use who had participated in the DMP, including individuals who initiated CGM during the study period as well as those who had initiated prior to January 1, 2022.

### Chart Reviews

Structured EHR review captured demographics, insurance type, diabetes regimen, number of DMP visits, and HbA1c values. For time-varying variables (age, insurance type, diabetes regimen, and number of DMP visits), the most recent value documented during the study period was used. Age at diagnosis was calculated using the documented year of diabetes diagnosis in the electronic health record and the patient’s age and was treated as a continuous variable in regression analyses. CGM start dates and device types were obtained from the EHR and cross-verified with Dexcom and Libre manufacturer databases, which were used when discrepancies occurred.

### Surveys

Telephone surveys were conducted in English or Spanish and included open-ended questions on CGM likes and dislikes, quantitative items on recent wear time and scanning frequency, and 7 Likert-scale items assessing engagement behaviors. Although device start and end dates were verified using manufacturer portals, detailed cloud-based adherence metrics were not systematically extracted. Recent CGM use and scanning frequency were therefore assessed via patient self-report as part of the structured survey. Responses were recorded in REDCap, and participants received a $50 gift card.

### Outcome Measures

Primary outcomes included: (1) CGM uptake, (2) time to 1% reduction in HbA1c, (3) patient-reported perceptions of CGM use, and (4) CGM engagement behaviors. Uptake was defined as any documented CGM use in the EHR or manufacturer database. A ≥1% HbA1c reduction was selected based on prior randomized trials of CGM use.^11-13^

### Statistical Analysis

Descriptive statistics summarized demographic and clinical characteristics overall and by CGM use, based on values documented at the final DMP visit during the study period. Baseline HbA1c was the first value recorded during the study period. Differences between ever and never users were assessed with chi-square tests for categorical variables and *t*-tests for continuous variables. CGM initiation trends were summarized in half-year intervals by insurance type. Among CGM users, total duration of documented use was summarized using medians and interquartile ranges.

Time to achieving a ≥1% HbA1c reduction was evaluated using Kaplan-Meier curves and log-rank tests. Time zero was defined as the baseline HbA1c measurement. For CGM new users (defined as individuals who initiated CGM during the study period), baseline HbA1c was the most recent value prior to CGM initiation. For never users, baseline HbA1c was the first available measurement during the study period. Participants were followed from baseline until the first HbA1c demonstrating a ≥1% reduction or were censored at the date of their last recorded HbA1c measurement within the study window. Individuals who initiated CGM prior to January 1, 2022, were excluded from the time-to-event analysis because a study-period pre-initiation baseline HbA1c was not available. Cox proportional hazards models estimated unadjusted and adjusted hazard ratios, adjusting for baseline HbA1c, age at diagnosis, language, and insurance type. Sensitivity analyses were conducted additionally adjusting for medication regimen and number of DMP visits to assess the robustness of the primary model estimates.

Open-ended responses were analyzed using an inductive thematic approach. A clinician-researcher with diabetes expertise coded responses and grouped similar concepts into categories, which were summarized as frequencies. Engagement measures were analyzed descriptively.

Analyses were conducted in RStudio version 4.4.0 and Stata version 18.0. The study protocol was reviewed and approved by an academic institutional review board, and verbal informed consent was obtained from survey participants.

## Results

A total of 669 adult patients received care in the Diabetes Management Program (DMP) during the study period, of whom 256 met eligibility criteria and were included in the chart review analysis (Supplemental eFigure 1). Of these, 126 patients (49%) were classified as CGM “ever users,” defined as individuals who initiated a CGM at any point during or prior to the study period, and 130 (51%) were categorized as “never users.” Among CGM ever users, 78% used intermittently scanned CGM (FreeStyle Libre 14-day and FreeStyle Libre 2) and 22% used real-time CGM (FreeStyle Libre 3, Dexcom G6, and Dexcom G7). Of the 126 CGM ever users, 82 (65%) completed the patient survey assessing perceptions of CGM use. CGM engagement behaviors were further evaluated among the 51 respondents (60%) who were actively using a CGM at the time of survey administration.

Demographic characteristics and most recent documented clinical variables during the study period are shown in Table 1. Overall, 48% of patients were aged 50 to 64 years, 58% were female, and 87% identified as Latino or Hispanic, with Spanish as the primary language for 75%. Most were insured through Medicaid (62%), and 55% were treated with basal insulin with or without non-insulin agents. Baseline glycemic control was generally suboptimal, with 41% of patients having an HbA1c ≥10%, indicating a high burden of uncontrolled diabetes in this population.

**Table 1.** Demographic and Clinical Characteristics of Chart Review Population.

Variable	Ever (n = 126)	Never (n = 130)	Total (n = 256)	*P*-value
Age				.361
<50	15%	21%	18%	
50-64	52%	44%	48%	
65+	33%	35%	34%	
Birth sex				.585
Female	60%	56%	58%	
Male	40%	44%	42%	
Race/ethnicity				.314
Latino/Hispanic	84%	91%	87%	
White	9%	4%	7%	
Black/African American	5%	4%	4%	
Asian	2%	1%	2%	
Primary language				.112
English	29%	18%	24%	
Spanish	70%	80%	75%	
Other	1%	2%	1%	
Insurance type				<.001
Medicaid	83%	41%	62%	
Medicare	14%	24%	19%	
No insurance	2%	30%	16%	
Commercial insurance	1%	5%	3%	
Medication regimen				.018
Basal/Bolus ± non-Insulin Agents	41%	28%	35%	
Basal ± non-insulin agents	46%	63%	55%	
U500+	4%	0%	2%	
NPH/R+	1%	2%	1%	
Non-insulin drugs only	8%	7%	7%	
# of DMP visits				<.001
2-5 visits	22%	43%	33%	
>5 visits	78%	57%	67%	
Baseline HbA1c				.036
<7%	3%	5%	4%	
7-7.9%	12%	18%	15%	
8-8.9%	17%	28%	23%	
9-9.9%	22%	13%	17%	
10% or greater	46%	36%	41%	
CGM type				—
Intermittently scanned (isCGM)	78%	—	—	
Real-time (rtCGM)	22%	—	—	
Time on CGM, months, median (IQR)	8.2 (4.6-13.2)	—	—	—

When stratified by CGM use, there were no statistically significant differences in age, sex, race and ethnicity, or primary language between ever and never users (Table 1). However, several meaningful differences emerged across clinical and program-related factors. CGM users were substantially more likely to be insured through Medicaid (83% vs 41%; *P* < .001). Insulin regimen patterns also differed, with CGM users more frequently prescribed basal-bolus insulin with or without non-insulin agents (41% vs 28%; *P* = .018). Program engagement was higher among CGM users, with 78% having more than 5 DMP visits (*P* < .001). CGM users also had higher baseline HbA1c values, with 46% having an HbA1c ≥10% compared with 36% of never users (*P* = .036).

**Figure 1.**
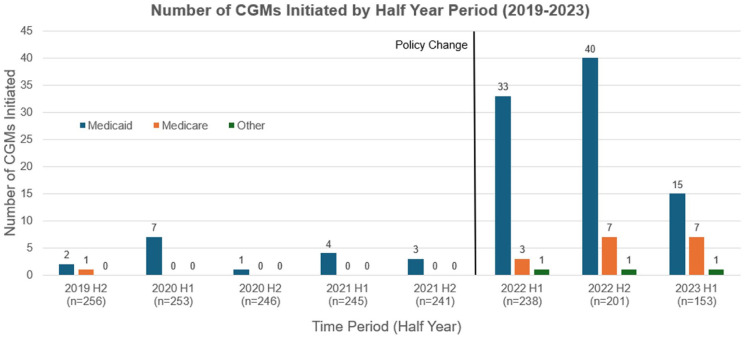
Number of continuous glucose monitors (CGMs) initiated by half year period from 2019 to 2023, stratified by insurance type. Prior to the January 2022 Medicaid coverage expansion (left of vertical line), CGM initiations were low across insurance groups. Numbers below each half-year represent the number of adult patients with type 2 diabetes eligible for CGM who had not previously initiated CGM at the start of that interval. Following the policy change (right of vertical line), CGM initiations increased, particularly among Medicaid-insured patients. The Other category included commercially insured and uninsured patients who obtained CGMs through grant support or self-payment.

Trends in CGM initiation by insurance category are shown in Figure 1. Prior to the January 2022 Medicaid coverage expansion, CGM starts were low across all groups, ranging from 1 to 7 starts per half-year among Medicaid patients, 0 to 1 among Medicare patients, and 0 among patients classified as “Other.” The “Other” category included individuals with commercial insurance and those who were uninsured; some uninsured patients later obtained CGMs through grant support or self-payment. Following the policy change, CGM uptake increased markedly, particularly among Medicaid-insured patients. In the first half of 2022, Medicaid CGM starts increased to 33, compared with 3 among Medicare patients and 1 in the other insurance category. This pattern peaked into the second half of 2022, with 40 Medicaid starts, 7 Medicare starts, and 1 Other start.

Among the 126 CGM ever users, median total documented CGM use was 8.2 months (IQR = 4.6-13.2 months). At the end of the study period, 96 of 126 users (76%) remained active CGM users. Among these current users, median duration of use was 10.3 months (IQR = 6.6-14.0 months), whereas among past users (n = 30), median duration was 1.8 months (IQR = 0.9-4.6 months).

**Figure 2.**
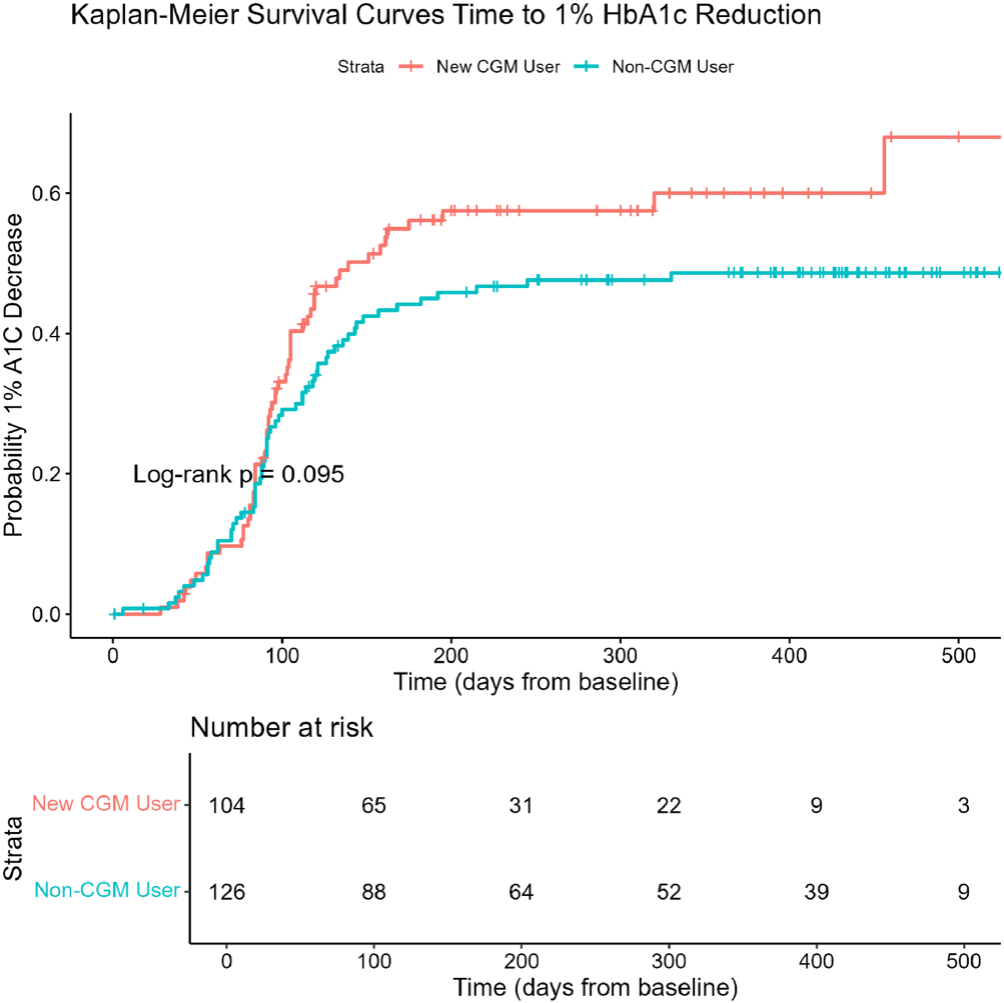
Kaplan-Meier survival curves comparing time to 1% HbA1c reduction among CGM new users vs never users. The curves display the cumulative probability of achieving at least a 1% decrease in HbA1c from baseline for CGM new users (red) and never users (blue). Time zero was defined as the baseline HbA1c measurement (most recent value prior to CGM initiation for new users and first available value during the study period for never users). Participants were followed until the first HbA1c demonstrating a ≥1% reduction or were censored at their last recorded HbA1c measurement within the study window. Tick marks indicate censored observations. Numbers at risk at selected time points are shown below the *x*-axis. The curves suggest a trend toward earlier ≥1% HbA1c reduction among CGM users (log-rank *P* = .095).

Time-to-event analysis for glycemic improvement is shown in Figure 2. The analytic sample included 230 patients, after excluding ever users who initiated CGM prior to January 1, 2022, and individuals without eligible baseline or follow-up HbA1c measurements during the study period. Kaplan-Meier curves demonstrated a trend toward CGM new users achieving a ≥1% reduction in HbA1c sooner than never users, although this difference did not reach statistical significance (log-rank *P* = .095). In the unadjusted Cox proportional hazards model, CGM new users had a non-significant higher likelihood of achieving a ≥1% HbA1c reduction (HR = 1.40; 95% CI: 0.97-2.01; *P* = .070) compared with never users (Table 2). There was no change in the association after adjustment for baseline HbA1c, language, age at diagnosis, and insurance type (adjusted HR = 1.31; 95% CI: 0.87-1.97; *P* = .202). Baseline HbA1c was significantly associated with time to achieving a ≥1% reduction in all models. In sensitivity analyses additionally adjusting for medication regimen and number of DMP visits, hazard ratio estimates were not materially changed.

**Table 2.** Cox Proportional Hazards Models of 1% HbA1C Reduction.

	Model 1: Unadjusted		Model 2		Model 3: Fully adjusted	
Variable	HR (95% CI)	*P*	HR (95% CI)	*P*	HR (95% CI)	*P*
CGM use						
Never	Ref		Ref		Ref	
New	1.40 (0.97,2.01)	.070	1.37 (0.95,1.98)	.090	1.31 (0.87,1.97)	.202
Baseline HbA1c, *per 1 point*	1.39 (1.29,1.50)	<.001	1.42 (1.31,1.54)	<.001	1.42 (1.31,1.54)	<.001
Language						
English			Ref		Ref	
Non-English			1.14 (0.68,1.92)	0.616	1.15 (0.67,1.97)	.620
Age at Diagnosis, *per 1 Year*			1.02 (1.00,1.04)	0.046	1.02 (1.00,1.04)	.105
Insurance						
Commercial					Ref	
Medicaid					1.39 (0.33,5.79)	.655
Medicare					1.34 (0.31,5.84)	.701
No Insurance					1.22 (0.28,5.40)	.790

Among CGM users who completed the survey, the most frequently cited reasons for liking CGMs were improved glucose management and convenience (Supplemental eFigure 2A). Additional benefits included reduced fingerstick burden and hypoglycemia-prevention features. Reasons for disliking CGMs were more varied (Supplemental eFigure 2B). Many participants reported no dislikes, while commonly cited concerns included physical discomfort or skin issues, alerts or information overload, and device-related challenges.

Among the 82 CGM users who completed the survey, 68% reported wearing their CGM every day over the past 2 weeks, while 20% reported no use during that period. Most respondents (85%) reported scanning their CGM or viewing their glucose values at least 3 times per day. Self-reported engagement behaviors among current users are shown in Table 3. Reviewing glucose trends and using CGM data to understand food-related effects were the most commonly reported behaviors, with 61% and 78% of participants, respectively, reporting they engaged in these activities “sometimes,” “often,” or “always.” A similar proportion (63%) reported using their CGM to prevent low blood sugars during exercise. Fewer respondents reported frequent CGM-guided insulin decisions: 41% reported modifying their insulin dose or timing based on trend information at least “sometimes,” and 20% reported taking extra insulin in response to high readings. Use of app-based notetaking was limited, with 26% reporting they used this feature “sometimes,” “often,” or “always.”

**Table 3.** Self-Reported CGM Engagement Behaviors Among Current Users.

Question (N = 51)	Never (%)	Rarely (%)	Sometimes (%)	Often (%)	Always (%)
How often do you look at your sugar trends or CGM reports?	29	10	24	12	25
How often do you look at your CGM data to determine how certain foods impact your sugar?	12	10	39	14	25
How often do you use your CGM to prevent low sugars when you exercise?	23	14	23	18	22
How often do you modify what you eat based on the information presented on your CGM?	16	18	23	16	27
How often do you modify your insulin dose or timing of insulin based on the information you see on your CGM? (for example, the direction of your trend arrow, or if sugar is too high or too low)	53	6	17	14	10
How often do you take extra insulin if your CGM tells you that your sugar is too high?	72	8	14	6	0
How often do you use your CGM reader or app to make notes about what you eat, or when you give insulin or exercise?	68	6	14	4	8

## Discussion

Our study examined CGM uptake, glycemic outcomes, patient-reported experiences and engagement behaviors among adults with type 2 diabetes receiving care in a safety-net setting following California’s 2022 Medicaid CGM coverage expansion. We found that CGM initiation increased substantially after the policy change, particularly among Medicaid-insured patients, suggesting that addressing insurance-related barriers can meaningfully expand access in underserved populations.

Prior studies have consistently shown that publicly insured and lower-income patients face substantial barriers to accessing CGMs, including more restrictive coverage criteria and lower prescribing rates.^14-17^ Our findings build on this work by demonstrating that a statewide Medicaid policy change can produce a rapid and meaningful increase in CGM initiation within a safety-net population. The policy change occurred within a clinical environment offering same-day CGM access, bilingual staff, and structured onboarding support, which may have amplified its impact. This combination of coverage expansion and program-level infrastructure may represent a scalable model for other safety-net systems.

Although the association between CGM use and time to achieving a ≥1% HbA1c reduction did not reach statistical significance, the trend toward improved glycemic outcomes among CGM new users aligns with findings from randomized trials and real-world studies of adults with insulin-treated type 2 diabetes.^10-13^ Recent observational analyses have further demonstrated clinically meaningful HbA1c reductions following CGM initiation, including large health system and national studies showing improved glycemic control and reduced acute metabolic events.^20,21^ The lack of a statistically significant difference in our cohort may reflect several factors, including modest sample size and variability in follow-up duration. In addition, both CGM users and never users received care within a structured Diabetes Management Program that provides frequent follow-up and medication titration, which may have led to glycemic improvements in both groups and attenuated between-group differences. Although adjustment for DMP visit frequency did not materially change the hazard ratio estimates, DMP engagement may plausibly represent part of the mechanism linking CGM initiation to glycemic improvement and was therefore not included in the primary model. In usual primary care settings with less intensive support, the effect of CGM on glycemic outcomes may be more pronounced. Nevertheless, the direction of the association, coupled with high patient-reported acceptability, supports the potential value of integrating CGMs into routine care for adults with poorly controlled type 2 diabetes.

Patient-reported experiences offered important new insights into how adults with type 2 diabetes in a safety-net setting interact with CGMs. Previous studies, largely conducted among privately insured or specialty clinic populations and many with type 1 diabetes, have highlighted convenience, real-time feedback, and reduced need for fingersticks as key advantages, and documented common challenges including skin irritation, alarm fatigue, and device burden.^23-28^ In our safety-net cohort, users similarly identified convenience, immediate glucose feedback, and reduced fingerstick burden as major benefits, while only some echoed familiar concerns (skin issues and information overload). Engagement behaviors in our study showed that although CGM users frequently check glucose values, few patients consistently use trend data to guide insulin dosing or leverage app-based features for self-management. These patterns in a publicly insured, resource-constrained population underscore that expanding access is necessary but not sufficient; tailored education may be particularly important in safety-net settings to help patients translate CGM data into effective therapeutic decisions.

This study should be interpreted in the context of several limitations. First, it was conducted within a single FQHC with a structured Diabetes Management Program. This model may limit generalizability to clinics with fewer resources, but it offers important insight into how policy change can translate into practice when paired with supportive infrastructure. Second, the retrospective nature of the EHR data limited our ability to standardize the timing of CGM initiation and HbA1c assessments. Consequently, differences in when these measures occurred across patients may have introduced variability in both exposure and outcome ascertainment. In addition, we did not have access to device-level CGM data and therefore could not assess metrics such as time in range or glycemic variability. Although total duration of CGM use was calculated using manufacturer portal and EHR cross-verification, we did not systematically extract detailed adherence metrics or assess continuous day-to-day wear. In addition, eligibility was defined based on any insulin use during the study period. Some patients were later transitioned off insulin, and we did not reassess insulin status over time or evaluate whether changes in therapy affected ongoing CGM eligibility or use. This may have introduced differences in clinical status during follow-up that were not fully accounted for. Third, although we adjusted for key covariates, the analysis of HbA1c reduction may have been underpowered to detect modest differences between groups, given variability in baseline HbA1c values and follow-up duration. Finally, survey responses relied on self-reported experiences and may be subject to recall bias. Additionally, survey respondents included individuals who initiated CGM both before and after the January 2022 policy expansion, which may have introduced heterogeneity in duration of use and prior experience that could influence reported perceptions and engagement behaviors. Despite these considerations, the study provides unique and timely evidence on how adults with type 2 diabetes in a safety-net setting initiate, use, and perceive CGMs following a major Medicaid coverage reform, an area for which data have been limited.

## Conclusion

In conclusion, this study demonstrates that Medicaid policy reform can substantially expand access to CGM technology in safety-net settings, particularly when paired with supportive clinical infrastructure. Although differences in time to glycemic improvements did not reach statistical significance, high uptake and positive patient-reported experiences suggest meaningful potential for benefit. Variability in engagement behaviors highlights the need for ongoing education and tailored support. Future research should evaluate long-term outcomes and strategies to optimize CGM integration across diverse care environments.

## Supplemental Material

sj-docx-1-jpc-10.1177_21501319261433345 – Supplemental material for Impact of Medicaid Continuous Glucose Monitor Expansion on Uptake, Glycemic Outcomes, and Engagement Among Adults With Type 2 Diabetes in a Federally Qualified Health Center Supplemental material, sj-docx-1-jpc-10.1177_21501319261433345 for Impact of Medicaid Continuous Glucose Monitor Expansion on Uptake, Glycemic Outcomes, and Engagement Among Adults With Type 2 Diabetes in a Federally Qualified Health Center by Walter Solorzano, Ligaya Docena Scarlett, Giuliana Perini Villanueva, Kathyana Santiago Mangual, Cynthia Santana, Bryan Escobar, Angelique Rubio, Nicholas J Jackson, Marielle Tavares, Joseph Borrell, Beatrice Brumley, Tannaz Moin and Estelle Everett in Journal of Primary Care & Community Health

## References

[1] Beck RW Riddlesworth TD Ruedy K , et al. Effect of continuous glucose monitoring on glycemic control in adults with type 1 diabetes using insulin injections: the DIAMOND randomized clinical trial. JAMA. 2017;317(4):371-378. doi:10.1001/jama.2016.19975 28118453

[2] Kwon SY Park JH Lee SA , et al. Advances in continuous glucose monitoring: clinical implications. Endocrinol Metab. 2022;37(1):25-36. doi:10.3803/EnM.2022.1389

[3] Prahalad P Addala A Buckingham BA Wilson DM Maahs DM. Sustained continuous glucose monitor use in low-income youth with type 1 diabetes following insurance coverage supports expansion of continuous glucose monitor coverage for all. Diabetes Technol Ther. 2018;20(9):632-634. doi:10.1089/dia.2018.0204 30020810 PMC6421989

[4] Miller KM Beck RW Foster NC Maahs DM. HbA1c levels in type 1 diabetes from early childhood to older adults: a deeper dive into the influence of technology and socioeconomic status on HbA1c in the T1D exchange clinic registry findings. Diabetes Technol Ther. 2020;22(9):645-650. doi:10.1089/dia.2019.0393 31905008 PMC7640747

[5] Gilbert TR Noar A Blalock O Polonsky WH. Change in hemoglobin A1c and quality of life with real-time continuous glucose monitoring use by people with insulin-treated diabetes in the landmark study. Diabetes Technol Ther. 2021; 23(S1):S35-S39. doi:10.1089/dia.2020.0666 PMC7957368 33470882

[6] Vigersky RA Fonda SJ Chellappa M Walker MS Ehrhardt NM. Short- and long-term effects of real-time continuous glucose monitoring in patients with type 2 diabetes. Diabetes Care. 2012;35(1):32-38. doi:10.2337/dc11-1438 22100963 PMC3241321

[7] Ehrhardt NM Chellappa M Walker MS Fonda SJ Vigersky RA. The effect of real-time continuous glucose monitoring on glycemic control in patients with type 2 diabetes mellitus. J Diabetes Sci Technol. 2011;5(3):668-675. doi:10.1177/193229681100500320 21722581 PMC3192632

[8] Yoo HJ An HG Park SY , et al. Use of a real time continuous glucose monitoring system as a motivational device for poorly controlled type 2 diabetes. Diabetes Res Clin Pract. 2008;82(1):73-79. doi:10.1016/j.diabres.2008.06.015 18701183

[9] Zick R Petersen B Richter M Haug C ; SAFIR Study Group. Comparison of continuous blood glucose measurement with conventional documentation of hypoglycemia in patients with Type 2 diabetes on multiple daily insulin injection therapy. Diabetes Technol Ther. 2007;9(6):483-492. doi:10.1089/dia.2007.0230 18034602

[10] Lind N Christensen MB Hansen DL Norgaard K. Comparing continuous glucose monitoring and blood glucose monitoring in adults with inadequately controlled, insulin-treated type 2 diabetes (Steno2tech study): a 12-month, single-center, randomized controlled trial. Diabetes Care. 2024;47(5):881-889. doi:10.2337/dc23-2194 38489032

[11] Martens T Beck RW Bailey R , et al. Effect of continuous glucose monitoring on glycemic control in patients with type 2 diabetes treated with basal insulin: a randomized clinical trial. JAMA. 2021;325(22):2262-2272. doi:10.1001/jama.2021.7444 34077499 PMC8173473

[12] Lever CS Williman JA Boucsein A , et al. Real time continuous glucose monitoring in high-risk people with insulin-requiring type 2 diabetes: a randomised controlled trial. Diabet Med. 2024;41(8):e15348. doi:10.1111/dme.15348 38758653

[13] Kim JY Jin SM Sim KH , et al. Continuous glucose monitoring with structured education in adults with type 2 diabetes managed by multiple daily insulin injections: a multicentre randomised controlled trial. Diabetologia. 2024;67(7):1223-1234. doi:10.1007/s00125-024-06152-1 38639876

[14] Addala A Maahs DM Scheinker D Chertow S Leverenz B Prahalad P. Uninterrupted continuous glucose monitoring access is associated with a decrease in HbA1c in youth with type 1 diabetes and public insurance. Pediatr Diabetes. 2020; 21(7):1301-1309. doi:10.1111/pedi.13082 32681582 PMC8103618

[15] Addala A Auzanneau M Miller K , et al. A decade of disparities in diabetes technology use and HbA_1c_ in pediatric type 1 diabetes: a transatlantic comparison. Diabetes Care. 2021; 44(1):133-140. doi:10.2337/dc20-0257 32938745 PMC8162452

[16] Sheikh K Bartz SK Lyons SK DeSalvo DJ. Diabetes device use and glycemic control among youth with type 1 diabetes: a single-center, cross-sectional study. J Diabetes Res. 2018; 2018:5162162. doi:10.1155/2018/5162162 PMC6087575 30151393

[17] Lai CW Lipman TH Willi SM Hawkes CP. Racial and ethnic disparities in rates of continuous glucose monitor initiation and continued use in children with type 1 diabetes. Diabetes Care. 2021;44(1):255-257. doi:10.2337/dc20-1663 33177169

[18] Wallia A Agarwal S Owen AL , et al. Disparities in continuous glucose monitoring among patients receiving care in federally qualified health centers. JAMA Netw Open. 2024; 7(11):e2445316. doi:10.1001/jamanetworkopen.2024.45316 PMC11584923 39576644

[19] Anderson JE Gavin JR Kruger DF. Current eligibility requirements for CGM coverage are harmful, costly, and unjustified. Diabetes Technol Ther. 2020;22(3):169-173. doi:10.1089/dia.2019.0303 31596132 PMC7047118

[20] California Department of Health Care Services. Medi-Cal Rx: Transitioning Medi-Cal Pharmacy Services from Managed Care to Fee-for-Service Frequently Asked Questions. 2022. Accessed October 20, 2024. https://medi-calrx.dhcs.ca.gov/cms/medicalrx/static-assets/documents/faq/Medi-Cal_Rx_Transitioning_Medi-Cal_Pharmacy_Services_from_Managed_Care_to_FFS_FAQs.pdf

[21] Gilliam LK Parker MM Karter AJ. Dysglycemic events after initiation of intermittently scanned continuous glucose monitoring in patients with insulin-treated type 2 diabetes. Diabetes Technol Ther. 2025;27(8):663-668. doi:10.1089/dia.2025.0021 40238709

[22] Karter AJ Parker MM Moffet HH , et al. Association of real-time continuous glucose monitoring with glycemic control and acute metabolic events among patients with insulin-treated diabetes. JAMA. 2021;325(22):2273-2284. doi:10.1001/jama.2021.6530 34077502 PMC8173463

[23] Tanenbaum ML Hanes SJ Miller KM Naranjo D Bensen R Hood KK. “It’s a very big deal”: patient perspectives on real-time continuous glucose monitoring. J Diabetes Sci Technol. 2017;11(3):567-572. doi:10.1177/1932296817691301 28745099 PMC5505433

[24] Polonsky WH Hessler D. What drives the use of continuous glucose monitoring? J Diabetes Sci Technol. 2017;11(4):766-771. doi:10.1177/193229681769130428322063

[25] Messer LH Berget C Forlenza GP. A clinical review of barriers to continuous glucose monitoring use in youth and adults. J Diabetes Sci Technol. 2017;11(3):482-487. doi:10.1177/1932296816685580

[26] Akturk HK Tanenbaum ML Kuznetsov A , et al. Real-world evidence on continuous glucose monitoring use and engagement among adults with diabetes. Diabetes Technol Ther. 2021;23(S1):S21-S27. doi:10.1089/dia.2020.0665

[27] Ruedy KJ Parkin CG Riddlesworth TD , et al. Continuous glucose monitoring trend arrows: recommendations for clinical decision-making. Diabetes Technol Ther. 2014;16(3):155-160. doi:10.1089/dia.2013.0307

[28] Walker AF Edelman SV Kolb L , et al. Continuous glucose monitoring and health literacy: understanding patient-level factors that influence effective use. Diabetes Technol Ther. 2019;21(10):522-530. doi:10.1089/dia.2019.017131219349

