# Supplementary material for: Impact of Medicaid Continuous Glucose Monitor Expansion on Uptake, Glycemic Outcomes, and Engagement Among Adults With Type 2 Diabetes in a Federally Qualified Health Center
Source: J Prim Care Community Health. 2026 Apr 20;17:21501319261433345. doi: 10.1177/21501319261433345 (PMC13111895; doi:10.1177/21501319261433345)

**Appendix**

eFigure 1: CONSORT Diagram


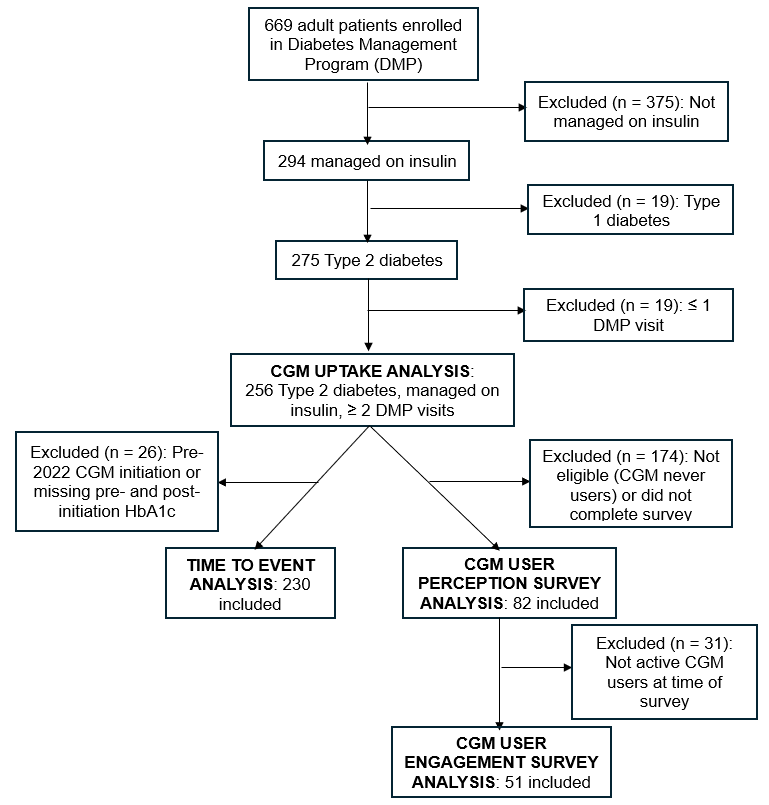


eFigure 2: Most Commonly Reported Reasons for Liking or Disliking CGMs (N = 82)


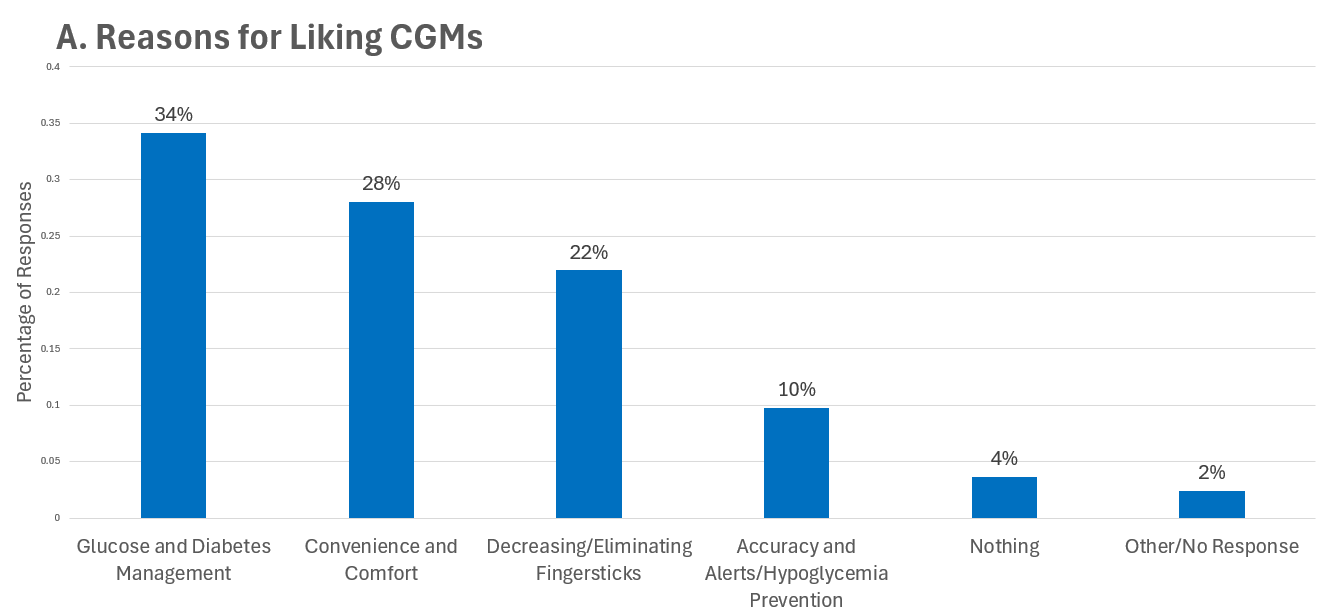

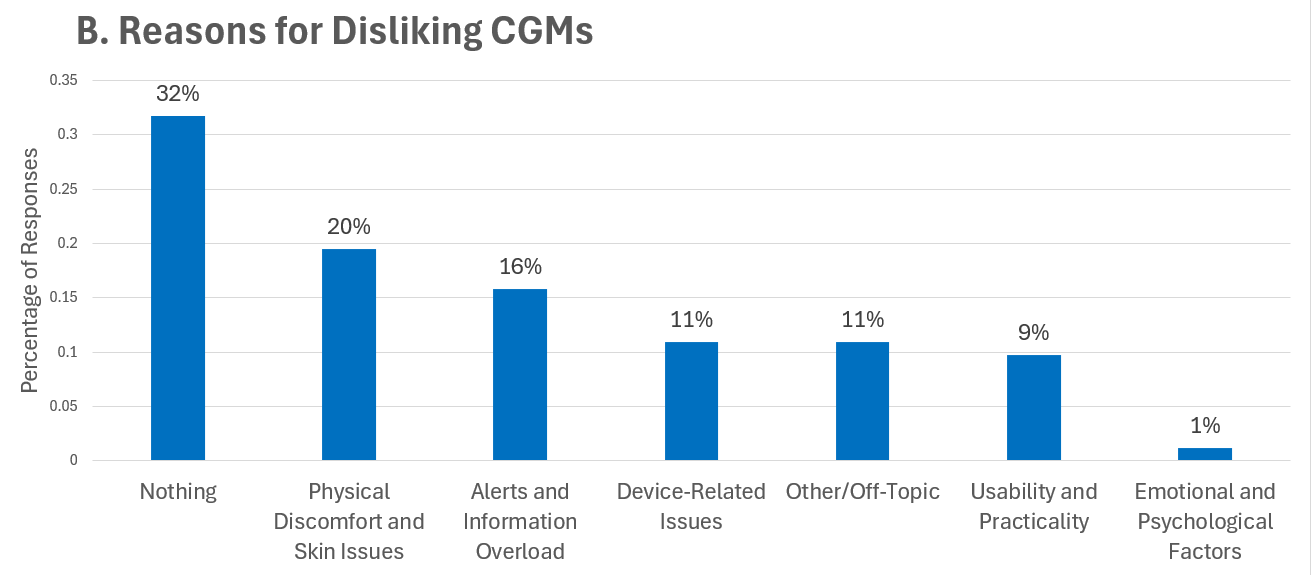

Supplement: sj-docx-1-jpc-10.1177_21501319261433345 – Supplemental material for Impact of Medicaid Continuous Glucose Monitor Expansion on Uptake, Glycemic Outcomes, and Engagement Among Adults With Type 2 Diabetes in a Federally Qualified Health Center [file sj-docx-1-jpc-10.1177_21501319261433345.docx]
